# The Hospital: A Medical Journal

**Published:** 1895-10-05

**Authors:** 


### The Hospital

AN INSTITUTIONAL JOURNAL OF

The Medical Sciences and Hospital Administration,

WITH

AN EXTRA NURSING SUPPLEMENT.

VOL. XIX.—No. 471]. [OCT. 5, 1895.

### THE HOSPITAL: A MEDICAL JOURNAL.

THE commencement of a new volume of THE HOSPITAL is a fitting occasion for making some reference to the growing importance of its position among the medical journals of this country, a position which is all the more secure from the fact that it has been gradually attained, not so much in response to any desire or ambition on our part, but because THE HOSPITAL met a want. It has been our duty at times to expose faults and to lay bare abuses in the management of various hospitals, but, amid such criticisms as we have felt called upon to make, we have never hesitated to admit, nay we have taken every occasion to assert, that hospitals are very much more than either mere receptacles for the sick, as was the older idea, or places of healing, as was the newer conception of their function. Besides all this they are and always must be the great centres of medical teaching, the great laboratories of medical investigation, and the highest representatives of the newest phases of the art of healing. It is doubtless owing to the universal recognition by medical men in all parts of the world of the fact that hospitals are centres of medical knowledge and starting points of medical progress that has led them to turn to THE HOSPITAL as the journal which gives them, more than any other, the information which is most valuable to them. At any rate, be the causes what they may, it has year by year become more clear that THE HOSPITAL, besides acting as the special organ of the institutions to represent which it was primarily founded, is circulating in constantly increasing numbers among members of the medical profession. Those who are connected with hospitals find it essential to them, giving them in short compass all the news of the work in other similar institutions, plans of new buildings which are being erected, descriptions of new appliances which are being found useful elsewhere, and the opinions of experts in the ever-varying details of hospital management. But they also find in it a weekly record of what is being done in them in medicine and surgery, and it is doubtless this which forms its special attraction to that ever-widening circle of readers, who, although not themselves in personal touch with hospital work, fly to it as a means of keeping themselves *en rapport* with what is going on in places which they justly look on as the fount of medical knowledge.

We on our part have found it impossible to hold back from the invitation thus offered to us by the medical profession, and thus it has happened that THE HOSPITAL has for some years past added to its older attributes those of a medical journal. This is a side of our work which we feel can now be usefully developed still further, and it is our intention to add in every possible way to the interest of the more essentially medical portion of THE HOSPITAL. Not only will its pages be found to contain contributions from leading authorities on such medical and surgical topics as are of most interest at the moment, but the medical aspect of current events will receive due comment. Every week also there will be found clinical records emanating from one hospital or another, and under the head of "Progress" a careful review will be given of all that is most recent in medicine, surgery, and the other branches of professional work. Medical men, however, cannot afford to isolate themselves from those who are occupied in other branches of research, and THE HOSPITAL will continue to offer to its readers comments on the books and the ideas which for the moment stir men's minds, things which might seem somewhat foreign to its title were it not true that we are all actors on the same stage and that none can duly play their part without attention to the playing of the rest. We have reason to believe that the Nursing Supplement published with the THE HOSPITAL every week is of more interest to medical men than some imagine. Nursing has become an essential part of all high-class medical work, and it is difficult for any practitioner to do the best possible for his patients unless he thoroughly appreciates the position nurses are now prepared to occupy and the sort of work they are prepared to do. This he can do by running his eye over the Nursing Mirror, which will give him a true reflection of the aims and feelings of nurses as a class, and of the standard of education which is common among them. We believe that knowledge so gained has not infrequently enabled practitioners to use nurses to the full in a way they would otherwise never have thought of doing.

Needless to say the management of a weekly journal covering such a wide field involves much labour on the editorial department. As, however, we have received encouragement, so have we not spared either labour or expense, having secured the collaboration of a considerable staff of medical men eminent in their various departments, so that our medical readers may feel assured that even the unsigned portions of The Hospital are produced by leaders in their own profession. The Hospital is now well recognised as a medical journal; the position has in a manner been thrust upon us—but we shall not turn back.

